# Yeast sexes: mating types do not determine the sexes in *Metschnikowia* species

**DOI:** 10.1093/femsyr/foae014

**Published:** 2024-04-17

**Authors:** Marc-André Lachance, Christopher Burke, Karen Nygard, Marc Courchesne, Alexander V Timoshenko

**Affiliations:** Department of Biology, University of Western Ontario, London, ON N6A 5B7, Canada; Department of Biology, University of Western Ontario, London, ON N6A 5B7, Canada; Okanagan Spirits Craft Distillery, 5204 24th St, Vernon, BC V1T 8×2, Canada; Biotron Experimental Climate Change Research Centre, University of Western Ontario, London, ON N6A 5B7, Canada; Department of Biology, University of Western Ontario, London, ON N6A 5B7, Canada; Biotron Experimental Climate Change Research Centre, University of Western Ontario, London, ON N6A 5B7, Canada; Department of Biology, University of Western Ontario, London, ON N6A 5B7, Canada

**Keywords:** Yeast, sexes, mating types, *Metschnikowia*, ascus mother cell

## Abstract

Although filamentous Ascomycetes may produce structures that are interpreted as male and female gametangia, ascomycetous yeasts are generally not considered to possess male and female sexes. In haplontic yeasts of the genus *Metschnikowia*, the sexual cycle begins with the fusion of two morphologically identical cells of complementary mating types. Soon after conjugation, a protuberance emerges from one of the conjugants, eventually maturing into an ascus. The originating cell can be regarded as an ascus mother cell, hence as female. We tested the hypothesis that the sexes, female or male, are determined by the mating types. There were good reasons to hypothesize further that mating type α cells are male. In a conceptually simple experiment, we observed the early stages of the mating reaction of mating types differentially labeled with fluorescent concanavalin A conjugates. Three large-spored *Metschnikowia* species, *M. amazonensis, M. continentalis*, and *M. matae*, were examined. In all three, the sexes were found to be independent of mating type, cautioning that the two terms should not be used interchangeably.

## Teaser

The sexes of three yeast species are not determined by their mating compatibility genes.

## Introduction

Yeasts are generally not thought to exhibit sexual dimorphism, where male and female structures might easily be recognized (Phaff et al. 1978). However, as with dimorphic species, where sexual offspring only arises from male-female pairs, yeast mating is not random. Compatibility in ascomycetous yeasts is controlled by a single locus for which two alleles, termed idiomorphs, determine the activation of genes that code for either the **a**- or the α-mating type-specific pheromones and their receptors (Wolfe and Butler 2022). In some filamentous Ascomycetes (Pezizomycotina), it is possible to distinguish male and female structures. Their differentiation is generally independent of mating types (Bennett and Turgeon 2016), although an association between the two has been reported in *Neurospora tetraspema* (Samils et al. 2013). In that species, several genes are co-expressed with the mating types. Mating type A strains favour sexual reproduction and a female bias, whereas mating type α strains show a tendency for asexual reproduction and a male bias.

Yeasts seldom differentiate into identifiable sexes. Exceptionally, McClelland et al. (2004) regarded the mating types α and **a** of the basidiomycetous yeast *Cryptococcus neoformans* as male and female, respectively, in view of the unidirectional movement of α-nuclei towards the dikaryon following conjugation. Heitman et al. (2013) also reported a tendency of **a**-cells to increase in size when exposed to the α-pheromone, which may allow recognition of the two mating types under the microscope. The larger mating type **a** cells represent cognates to the larger female gametes of oogamous species. In addition, consistent with the notion of sexes in *C. neoformans* is the report of uniparental inheritance of mitochondria by the presumed female (mating type **a**) cells (Xu et al. 2000). This notwithstanding, it is not common practice to designate yeast gametes as male or female, and some authors have gone as far as to assume that the notion of sexes is irrelevant to yeasts (Nieuwenhuis and Aanen 2012) or even to fungi in general (James 2023).

As always, clear definitions are key to fruitful discussion. Whether biological sexes are strictly binary as opposed to part of a spectrum is currently the object of considerable debate in some circles (Goymann et al. 2023, McLaughlin et al. 2023). The controversy is often fuelled by ambiguous terminology, where one fails to parse out secondary from primary sexual characteristics or to distinguish biological sexes from socially constructed genders. Reports of non-binary sexes in microorganisms, used as evidence for the non-binary nature of the sexes, are clear instances of equivocation, deliberate or otherwise. Specifically, the supposition that some fungi feature thousands of sexes (Kothe 1996), that a cellular slime mould has three sexes (Douglas et al. 2016), or that *Tetrahymena thermophila* is a seven-sex species (Yan et al. 2024) is in every case a patent misuse of “sexes” as a synonym of mating types, perhaps motivated by the wish to enhance the allure of journal article titles. Other examples exist (Iwasa and Sasaki 1987, Hurst 1996). Likewise, the suggestion that “the sexual identity of a strain is established by the mating-type locus” (Kim and Borkovich 2006) is not borne out by the source reference (Kronstad and Staben 1997) and is undoubtedly a case of imprecise use of the expression “sexual identity”. Similar criticisms apply to the use of “sex determination” by Ni et al. (2011) or “sex genes” by Idnurm et al. (2008).

It is of no help that the term sex is also used as a shorthand for sexual reproduction, regardless of whether it entails recognizable sexes. Equivocal terminology may be a sign of the times, as evidenced by a Google Ngram depicting a two-fold decrease in the use of the word “sexes” from its 20^th^ century maximum in 1977 to the year 2019, in contrast to the word “gender”, which experienced a 20-fold increase over the same period.

The frequent inclusion of motility in the definition of male gametes reflects a zoocentric bias that is pervasive in biology. Gamete size (anisogamy) is also a widely applied criterion, but similarly should be seen as overly restrictive, as it fails to account for clear cases of male-female polarity in isogamous organisms, as exemplified for *Spirogyra* species by Saunders (1931) or Takano et al. (2019). There, the label “female” is clearly understood to designate the conjugant that receives a nucleus donated by her male counterpart. We see the direction of movement of nuclei as a clear and widely applicable means of identifying male and female sexes. Recognition of the male gamete as that which donates its nucleus to a female gamete was explicit for *Cryptococcus neoformans* (McClelland et al. 2004).

Conjugation in some ascomycetous yeasts proceeds between a bud and its mother cell, a form of heterogamy. In those cases, bud formation, coupled with mitosis, culminates with the separation of two cytoplasms by a septum that is later dissolved, allowing the return of the bud nucleus to the mother cell, which then matures into an ascus containing meiotic spores. This was elegantly demonstrated by electron microscopy in *Debaryomyces hansenii* (Kreger-van Rij and Veenhuis 1975). The directionality of nuclear transfer from bud to mother cell thus makes the bud the male gamete, and the mother cell, the female gamete. These heterogamous buds are typically small, such that the notion of male-female polarity is further reinforced by a size dimorphism. Genetically, mother-bud conjugation implies that a species is homothallic, where all sister (mother cell and bud) nuclei are compatible while having identical genomes. Wolfe and Butler (2022) explained that homothallism arises either when every cell of a culture carries active forms of both mating type alleles, or when cells are capable of mating type switching. The former, termed primary homothallism, has been documented in several species, including *D. hansenii*, and so we must regard this as an instance where differentiation of the sexes is ontogenetic and independent of mating types. It is not inconceivable that sexes and mating types might be coupled in secondarily homothallic species, but this remains to be explored. In filamentous Ascomycetes, the formation of male and female structures (e.g. antheridia, ascogonia) appears to be governed by different sets of genes, although interactions between these and mating type genes are evident in some cases (Wilson et al. 2021). As already mentioned, some coupling has been noted in *Neurospora tetrasperma* (Samils et al. 2013).

The male-female polarity of bud-mother cell conjugation in *Debaryomyces* species is analogous to the sexes encountered in complex fungi or, for that matter, animals or plants, in the sense that the two sexes can be identified morphologically by size, the female conjugant being larger. However, conjugation in yeasts is often isogamous, proceeding between morphologically identical cells, as in the archetypal *Saccharomyces cerevisiae* (Herskowitz 1988). In such species, the recognition of an individual as male or female is problematic, such that one may argue that the concept of sexes simply does not apply. Absence of sexes does not preclude mate choice among strains of compatible mating types. In *Saccharomyces cerevisiae*, for example, mate choice is thought to affect evolutionary fitness (Pilpel et al. preprint).

**Figure 1.**
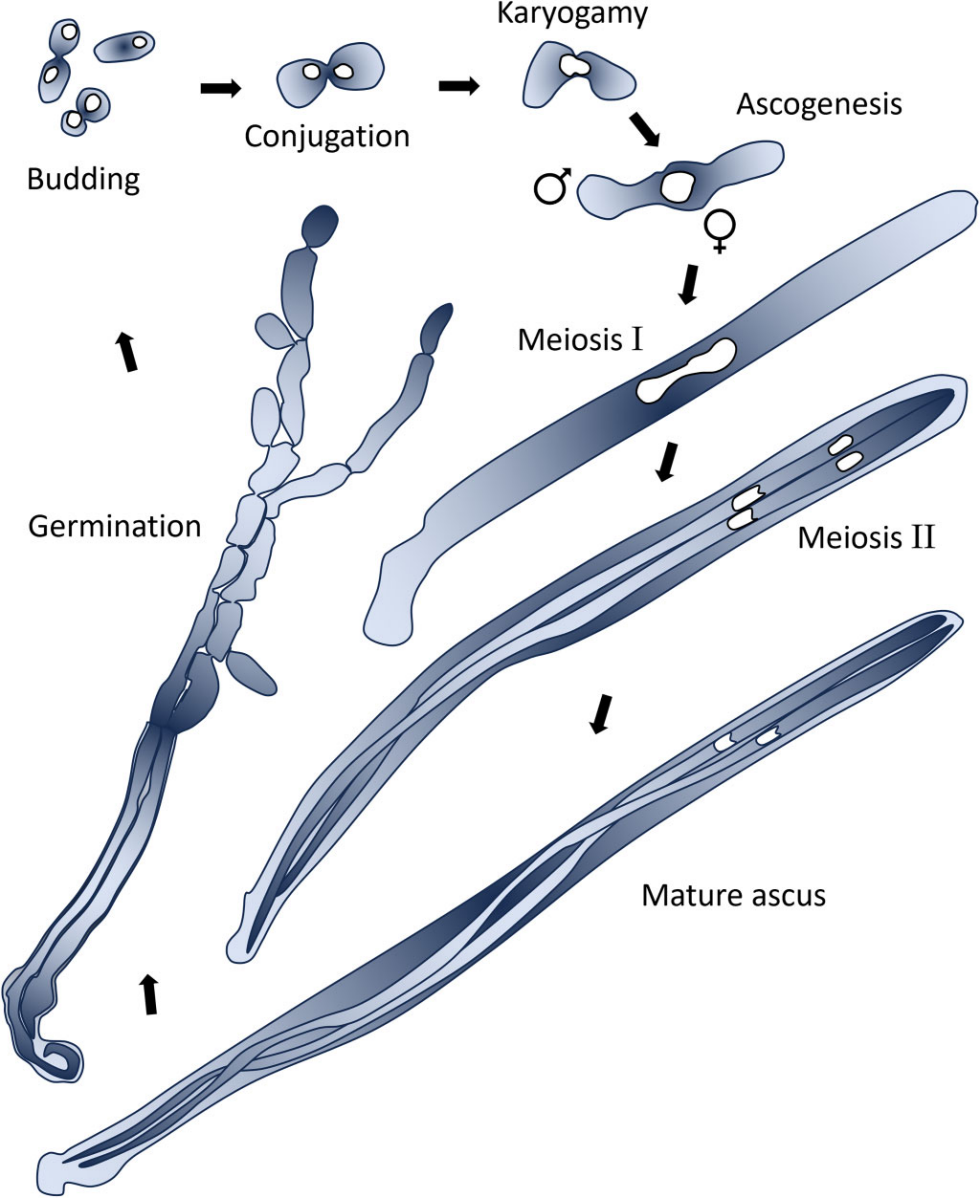
Sexual cycle of large-spored, haplontic *Metschnikowia* species. Haploid budding cells, conjugation (1-2 h), diploid zygote (2-3 h), and developing asci (5-72 h) of *M. hawaiiensis* traced from fluorescence micrographs of DAPI-stained material (see supplementary data Fig. S1). The conjugants are identifiable as male and female from the start of ascus development, where they differentiate, respectively, into a moiety devoid of its nucleus and a mother cell that produces a protuberance in which meiosis and ascospore development will take place. Germinating spores traced from a phase contrast micrograph of (unstained) *M. continentalis* (Marinoni and Lachance 2004). Here, the mature ascus, 18 h after transfer to fresh medium, showed cleavage of ascospores into budding cells.

The yeast genus *Metschnikowia* contains both diplontic and haplontic species that can engage in isogamous conjugation, prior to the transition from the haplophase to the diplophase. To the extent of the relevant genome data reviewed by Lee et al. (2018), all *Metschnikowia* species are heterothallic, each haploid strain harboring a single mating locus with either an α or an **a** idiomorph. In diplontic species, which are by necessity heterozygous at the mating locus, ascus formation is environmentally triggered (Lachance 2011); conjugation of haploid mating types is rarely observed but is presumed to occur in nature soon after ascospore germination. This is the case for the better-known species *M. bicuspidata*, a pathogen of crustaceans, *M. pulcherrima*, utilized in winemaking and biological control of fruit spoilage, and *M. reukaufii*, a major component of the nectar microbiota of many plants (Lachance 2016). Conjugation between germinating ascospores is of all evidence suppressed in haplontic *Metschnikowia* species, as they are invariably recovered in nature as haploid cultures, although they readily conjugate when mixed in the laboratory. Like all isogamous yeasts, haplontic *Metschnikowia* species initiate the sexual cycle by the fusion of morphologically identical cells. Importantly, they share the additional feature that the ascus clearly arises from one of the two conjugating cells, away from the point of fusion (Fig. 1). A male-female polarity thus exists, but unlike what is seen in anisogamous species, the two sexes become perceptible only after conjugation has taken place between identical gametes. The vestigial zygote consists of a clearly male, donor cell and a clearly female, ascus mother cell. Such a male-female polarity is also found in a few other genera, notably in a small clade of *Wickerhamiella* species typified by *W. occidentalis* (Lachance et al. 1998). Whether mating type alleles determine sexes in these yeasts is not known. Although the mating types of haplontic *Metschnikowia* species are easily determined by crossing a culture with reference strains of known mating types and observing zygote formation, which takes place within a few hours, the mating types of unconjugated cells in a mixed culture are indistinguishable.

Because of the asymmetry of the developing ascus, we hypothesized that male and female sexes in haplontic *Metschnikowia* species are controlled by the mating type locus. We further conjectured that α-cells would be male and **a**-cells female, given certain known differences between α and **a** mating pheromones. In *Metschnikowia* (Lee et al. 2018) and other yeast genera (Rogers et al. 2015), mating specificity is governed to a higher degree by the α-pheromone, which tends to diverge faster in evolutionary time. It follows that **a**-cells share a higher proportion of the fitness burden of mate selection, which is typically a female characteristic. Furthermore, **a**-pheromones of Ascomycetes differ by the presence of a farnesyl residue that reduces pheromone diffusibility. The farnesyl group does not appear to contribute to mate recognition itself (Gonçalves-Sá and Murray 2011), but it reduces the reach of the **a**-pheromone, and in so doing, limits the mating response of α-cells to those that are in close physical proximity to **a**-cells (at least in *Saccharomyces cerevisiae*; Anders et al. 2021), thus preventing a runaway feedback loop that would engender a rapid buildup of saturating concentrations of pheromones. Asymmetry in mating responses is prerequisite to sexual selection in isogamous species. Such asymmetries have been reported for a small clade of haplontic *Metschnikowia* species that are endemic to the Hawaiian archipelago, where they live in association with endemic nitidulid beetles (Lee et al. 2018). In those species, for example *M. hawaiiensis*, hybridization with moderate relatives (e.g. *M. continentalis*) only occurs in crosses between α-strains of the former and **a**-strains of the latter, and not in the reciprocal cross. These species have identical **a**-pheromones, but different α-pheromones. In preparation for the experiments to follow, a similar phenomenon was observed where hybrid (sterile) asci arise in crosses between α-strains of *M. amazonensis* and **a**-strains of the other two species, but not in the reciprocal crosses.

In *Saccharomyces cerevisiae*, the differences in diffusibility of the two pheromones, combined with the production by **a**-cells of protease Bar1, which specifically hydrolyses the α pheromone, results in differences in the mating behaviour of the two cell types (Banderas et al. 2016). Mating type **a** cells respond primarily to the ratio of α- over **a**-cells, whereas α-cells respond to the absolute density of **a**-cells. Additionally, Anders et al. (2021) identified two behavioural differences between the two cell types, namely, budding orientation and preferential conjugation tube development in α-cells. The latter might be construed as a male feature, although the authors refrained from doing so. By analogy to the formation of trichogynes by females of filamentous Ascomycetes (Kim and Borkovich 2006), caution is called for in assigning a sex polarity to conjugation tube development.

The polarity observed in *Cryptococcus neoformans* (McClelland et al. 2004), although creating a precedent for an association between mating types and sexes, does not entirely buttress our α-male/**a**-female hypothesis, as both pheromones of that species are farnesylated. Furthermore, the lack of detectable sequence identity observed in the peptide portion of ascomycete and basidiomycete pheromones casts doubt on whether they should be regarded as orthologues. Finally, at variance with what was observed in *C. neoformans*, an association between mating type and mitochondrial inheritance has not been observed in *Metschnikowia* species, including *M. continentalis* (Marinoni and Lachance 2004).

Alternatives to the α-male/**a**-female hypothesis in *Metschnikowia* species are (1) that α-cells are female or (2) that the sexes arise independently of mating types. An adaptationist explanation for either outcome would be more difficult to formulate. Any association between sexes and mating types, regardless of polarity, would however begin to shed light on the so-called non-sex genes found in the two mating type alleles of all species of the Metschnikowiaceae (Riley et al. 2016, Lee et al. 2018). In that family, mating loci contain, in addition to the mating genes *mat***a***1* and *mat***a***2*, or *matα1*, three genes that do not appear to bear on mating compatibility. Two of these non-collinear genes, *PIK1* (phosphatidylinositol kinase) and *PAP1* (poly(A) polymerase) are essential in several species including *Candida albicans* (like *Metschnikowia*, a member of the order Serinales, but in the stem family Debaryomycetaceae; Groenewald et al. 2023), but deletion of a third gene, *OBP1* (oxysterol binding protein), is not lethal (Srikantha et al. 2012). The genes are suspected to play a role in infectivity of *C. albicans* by affecting the permeability of biofilms and their susceptibility to antibiotics such as fluconazole, but the mechanism is not known.

Non-sex genes have been more appropriately named non-mating genes in a discussion of *Candida auris* (Muñoz et al. 2018), a member of the family Metschnikowiaceae. Were it demonstrated that *PIK1, PAP1*, and *OBP1* do indeed determine sex polarity, the expression “sex-determining genes” would indeed be a more accurate characterisation, emphasizing their distinct but complementary nature with respect to the *mat***a***1, mat***a***2*, and *matα1* transcription factor-encoding genes known to regulate the expression of mating genes.

To shed light on these matters, we labeled mating types of three *Metschnikowia* species with fluorescent concanavalin A (conA) conjugates and observed their arrangement during mating. Our hypotheses allow three mutually exclusive predictions: (1) that all cells identified as male will be of mating type α, (2) that all females will have mating type α, or (3) that the joint distribution of sexes and mating types will be random (50:50).

## Methods

### Yeast cultures

The strains were obtained from the yeast culture collection of the Department of Biology (formerly Plant Sciences) of the University of Western Ontario (UWOPS), where they are kept frozen in liquid nitrogen. They were selected to represent the two mating types of three closely or moderately related species for which draft genome sequences have been deposited.

### Mating and microscopy

Mating experiments were prepared from cultures grown on YM agar (Glucose 1%, peptone 0.5%, malt extract 0.3%, yeast extract 0.3%, agar 2%). Similar results were obtained from overnight cultures kept at 25°C or with up to two-week-old cultures kept at 15°C. For DAPI staining, crosses were performed by mixing small, equal amounts of compatible cells on corn meal agar (Difco, Detroit, MI, USA). Mixtures were observed periodically to identify the various stages of ascogenesis, from zygote formation (1-3 h), to ascus development (4-6 h) to maturation of the two ascospores (1–3 d). Suitably diluted, formaldehyde-fixed cells were applied to an agar block and transferred to a conA-coated coverslip, following the procedure of Streiblová and Hašek (1996). They were then stained with 2 µg.mL^−1^ 4’,6-diamidino-2-phenylindole (DAPI, Sigma Aldrich, St. Louis, MO, USA) as described by Balasubramanian et al. (1997) and observed under a Zeiss Axioimager A.1 fluorescence microscope with the DAPI filter cube (358/463 nm) and a 100x objective. The images were taken in both fluorescence and bright field modes with a high-resolution monochrome XCD-X700 CCD camera (Sony) using Northern Eclipse 8.0 software from Empix Imaging Inc. (Mississauga, ON, Canada).

Mating type-specific staining followed a modification of the protocol of Lockhart et al. (2003). Cells of each mating type were suspended in sterile water, centrifuged, and resuspended in 0.5 mg.mL^−1^ Alexa Fluor 488-conjugated conA (green/cyan) or Alexa Fluor 647-conjugated conA (red/magenta), both obtained from ThermoFisher Scientific, London, ON, Canada, and dissolved in 0.1 M NaHCO_3_. These procedures were carried out in the dark with a red headlamp. After 1 h at room temperature, the cells were twice centrifuged and resuspended in water. Twenty microlitres of the stained suspensions were mixed and inoculated onto Yeast Carbon Base (Difco) agar with 0.01% yeast extract (YCBY) and incubated at room temperature in the dark. Observations took place 5 to 7 h after mixing. Agar blocks carrying the mating mixtures were transferred, cells down, onto coverslips, in a drop of Citifluor antifadent mountant (Electron Microscopy Science) containing 100 µM calcofluor (Sigma). The preparations were observed in a Nikon Eclipse Ti2-E microscope (Nikon Canada Inc., Mississauga, ON, Canada) with a 60x oil immersion objective and a 1.5x intermediate magnification, at the Microscopy Centre of the Biotron, University of Western Ontario. For each image, a focal series was recorded at each appropriate excitation/emission wavelength for the three fluorophores (calcofluor: 378/432 nm; Alexa 488: 474/515 nm; Alexa 647: 635/680 nm). Images were deconvolved using NIS Elements imaging software (Nikon) version 5.42.03 and extended depth of focus images were created to yield two-dimensional images with all parts of the cells in focus. The three fluorescent colours were optimized manually to give the clearest interpretation of the location of the two conjugants relative to nascent asci. Although the observation of two different conjugation figures would be sufficient to reject hypotheses 1 or 2, the minimum of 21 conjugation figures observed for each species ensures a good fit to the binomial distribution, were hypothesis 3 to prevail. Reciprocal experiments with both combinations of Alexa Fluor conA conjugates and mating types were conducted to rule out any differential effects of the dyes on the mating outcome.

## Results

**Figure 2.**
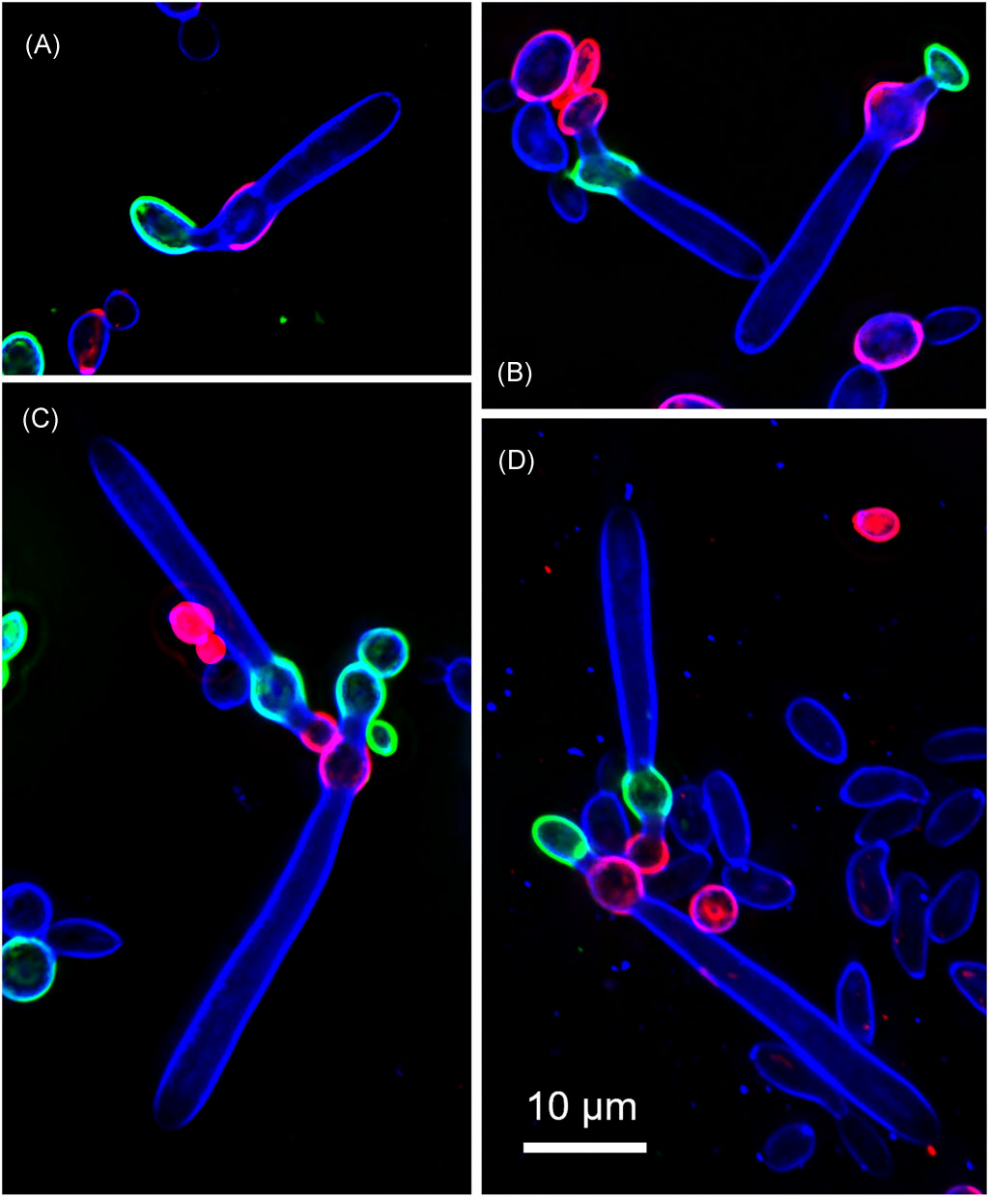
Random joint distribution of mating types and sexes in three species of *Metschnikowia, M. amazonensis* (A,B), *M. continentalis* (C), and *M. matae* (D). Alexa Fluor-labeled mating types (α = red/magenta, A,C or green/cyan B,D). Ascus mother cells (female) are those proximal to the developing asci (blue fluorescence of calcofluor) and the conjugated cells distal of the nascent asci are regarded as male. A complete set of images can be found in the supplementary data as Figs S2 to S5.

Figure 2 shows examples of the images recorded for the three species examined. Single cells or members of conjugated pairs in the zygotes clearly stained green or red. Fluorescent labeling of either mating type with either Alexa Fluor gave equivalent results. Developing asci, which arose after mating type-specific staining, acquired the blue fluorescence characteristic of calcofluor. Importantly, in the three species examined, asci grew out of either mating type in zygotes (Table 1). The numbers lie within the 95% confidence limits of a 50:50 binomial distribution, the largest spread, seen in *M. amazonensis*, sitting at 1.95 standard deviations from the expected mean of 0.5.

**Table 1.** Female and male sexes are independent of mating types. Strains of *Metschnikowia* species and results of crosses. The number of zygotes where mating types **a** or α became the female or males sexes is shown. Raw data can be found in the supplementary data, Figs S2 to S5. Superscripts identify type strains and allotypes, as applicable. UFMG are accession numbers in the culture collection of the Universidade Federal de Minas Gerais, Belo Horizonte, MG, Brazil.

Species	Strain number	Mating type	a♀/α♂	a♂/α♀
*M. amazonensis*	UFMG-CM-Y6309^T^	**a**	37	22
	UFMG-CM-Y6307^A^	α		
*M. continentalis*	UWOPS 96–173^T^	**a**	8	13
	UWOPS 95–402.1	α		
*M. matae*	UFMG-CM-Y395^T^	**a**	14	8
	UFMG-CM-Y-391^A^	α		

## Discussion

Interpretation of the numbers in Table 1 is straightforward. Were mating loci responsible for determining sex polarity, one would have obtained exclusively zygotes where all male conjugants were of the same mating type, be it α or **a**. However appealing, the hypothesis of interdependence of the sexes and the mating types must be rejected. Instead, the results support the notion that alleles of the mating locus have no direct bearing on which half of the zygote gives rise to the ascus. It follows that sexual polarity is independent, not only of the mating genes proper, but also of the so-called non-sex genes found in the mating loci of metschnikowiaceous yeasts, such that their renaming to sex-determining genes is not justified, although the term non-mating genes remains more appropriate. Sex determination across the living world is extremely diverse, but a large proportion of the research on this topic is focused on animals or plants (Tree of Sex Consortium 2014). The experiment described here, however simple, provides an important element towards our understanding of sex determination in yeasts, namely that sex polarity can be the result of the chance movement of the nascent diploid nucleus to one or the other parent cell, regardless of mating type. Our observations were limited to species of a single genus. It will be worthwhile to examine other cases where ascogenesis is asymmetrical, for example species of the distantly related genus *Wickerhamiella*, to obtain a better sense of generality. Importantly, the result highlights the need to exercise utter care in the choice of terminology surrounding sex, the sexes, sexual identity, sex determination, sex genes, or non-sex genes in discussions of these and other related topics such as mating compatibility or gender.

## Supplementary Material

foae014_Supplemental_Files
