# Supplementary material for: Yeast sexes: mating types do not determine the sexes in Metschnikowia species
Source: FEMS Yeast Res. 2024 Apr 17;24:foae014. doi: 10.1093/femsyr/foae014 (PMC11078162; doi:10.1093/femsyr/foae014)
Supplement: foae014_Supplemental_Files [file foae014_supplemental_files.zip › foae014.docx]

Fig. S1. DAPI-stained mating cells and asci of *Metschnikowia hawaiiensis* strains UWOPS 91‑745.2 and UWOPS 87-2203.2 used to trace the illustration in Fig. 1. A bright field image of the 48h ascus was used to trace the contour of the ascus. The scale bars show 5 µm.

Fig. S2. Images used to determine the mating type/sex distributions reported in Table 1. The values for *M. amazonensis* are the sum of those in this figure (22α♂:11α♀) plus those in Fig. S3. Here, mating type α cells were stained with the red/magenta fluorophore. Letters indicate figures used as evidence of female (F) or male (M) α-cells. Codes point to rows of images recorded in separate fields. All figures that could be interpreted as α♂ or α♀ were excerpted from each field. Separate photographs for each of the three fluorophores are available.

Fig. S3. Images used to determine the mating type/sex distributions reported in Table 1. The values for *M. amazonensis* are the sum of those in this figure (15α♂:11α♀) plus those in Fig. S2. Here, mating type α cells were stained with the green/cyan fluorophore. See Fig. S2 for other details.

Fig. S4. Images used to determine the mating type/sex distributions reported in Table 1 for *M. continentalis*. Mating type α cells were stained with the red/magenta fluorophore. See Fig. S2 for other details.

Fig. S5. Images used to determine the mating type/sex distributions reported in Table 1 for *M. matae*. Mating type α cells were stained with the green/cyan fluorophore. See Fig. S2 for other details.
