# Supplementary figures and images for: Yeast sexes: mating types do not determine the sexes in Metschnikowia species
Source: FEMS Yeast Res. 2024 Apr 17;24:foae014. doi: 10.1093/femsyr/foae014 (PMC11078162; doi:10.1093/femsyr/foae014)

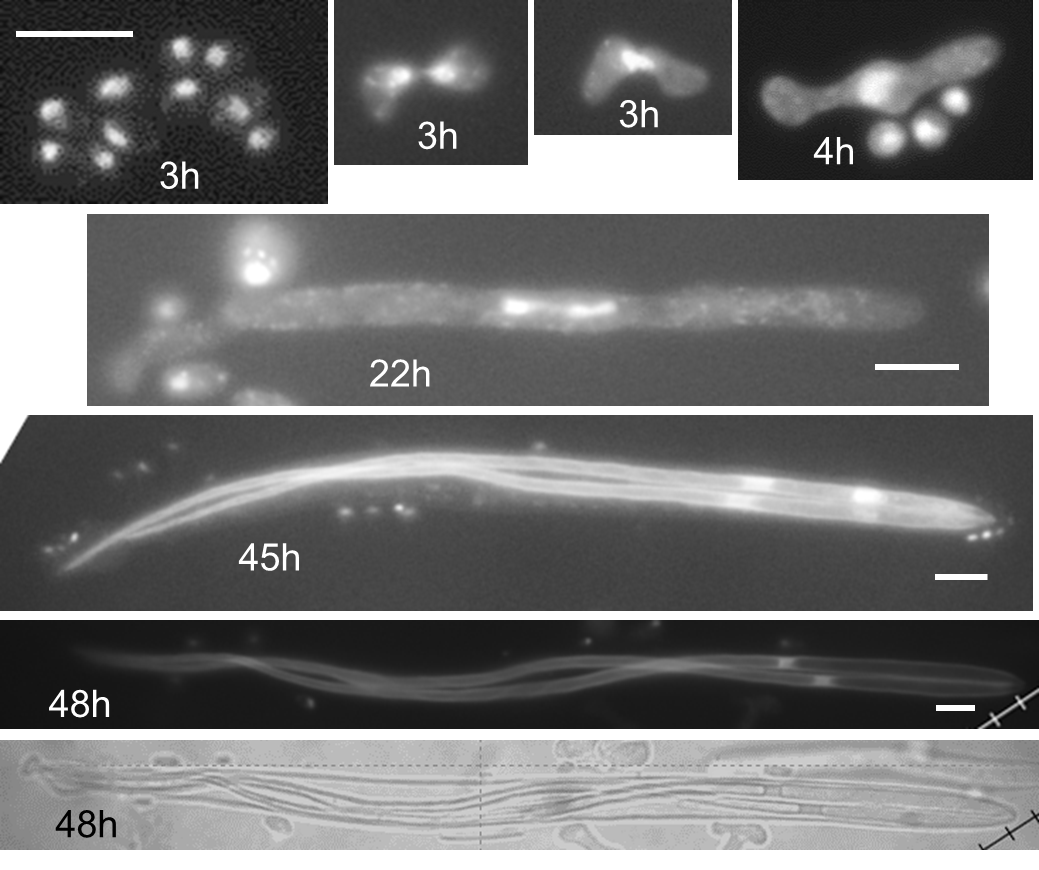

Supplement: foae014_Supplemental_Files [file foae014_supplemental_files.zip › Fig. S1 Supplementary data.tif]

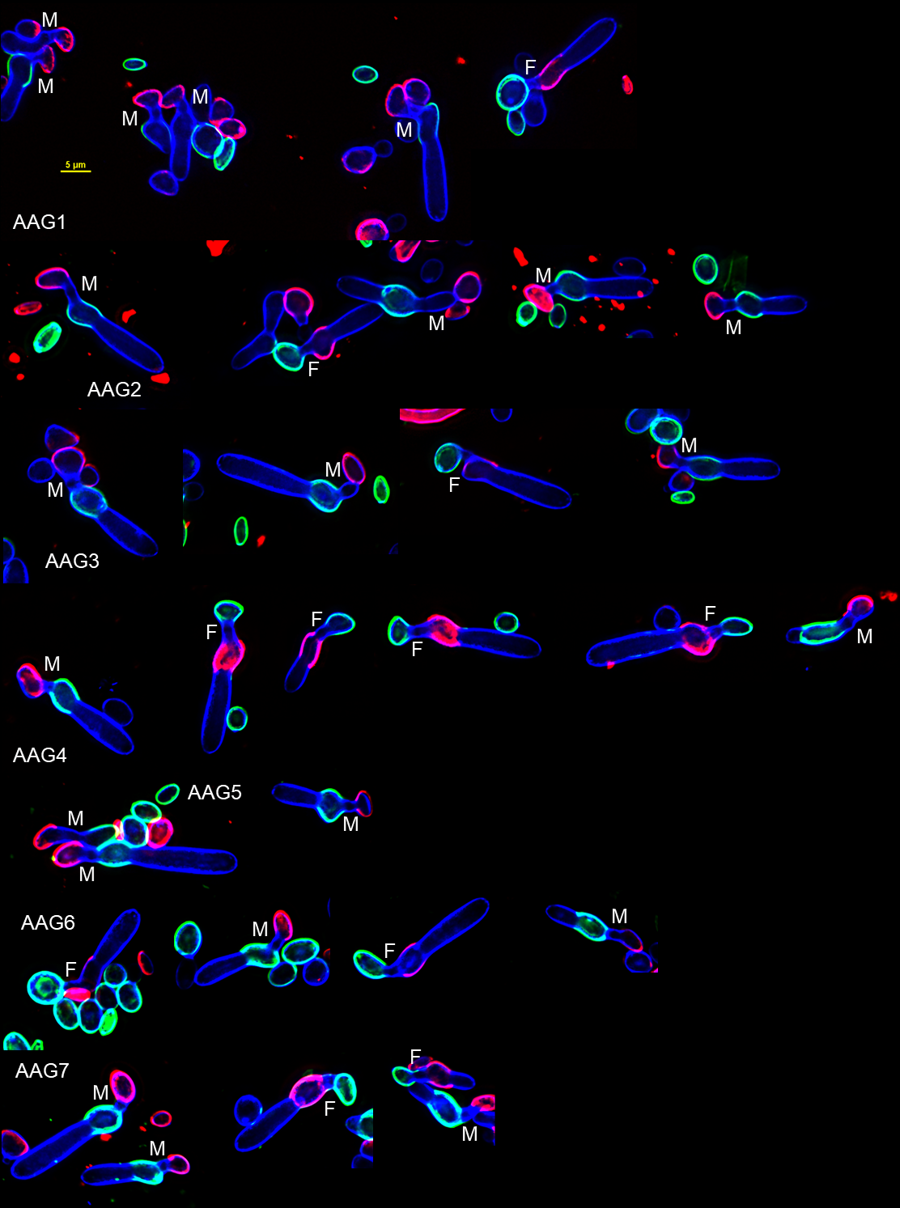

Supplement: foae014_Supplemental_Files [file foae014_supplemental_files.zip › Fig. S2 Supplementary data.tif]

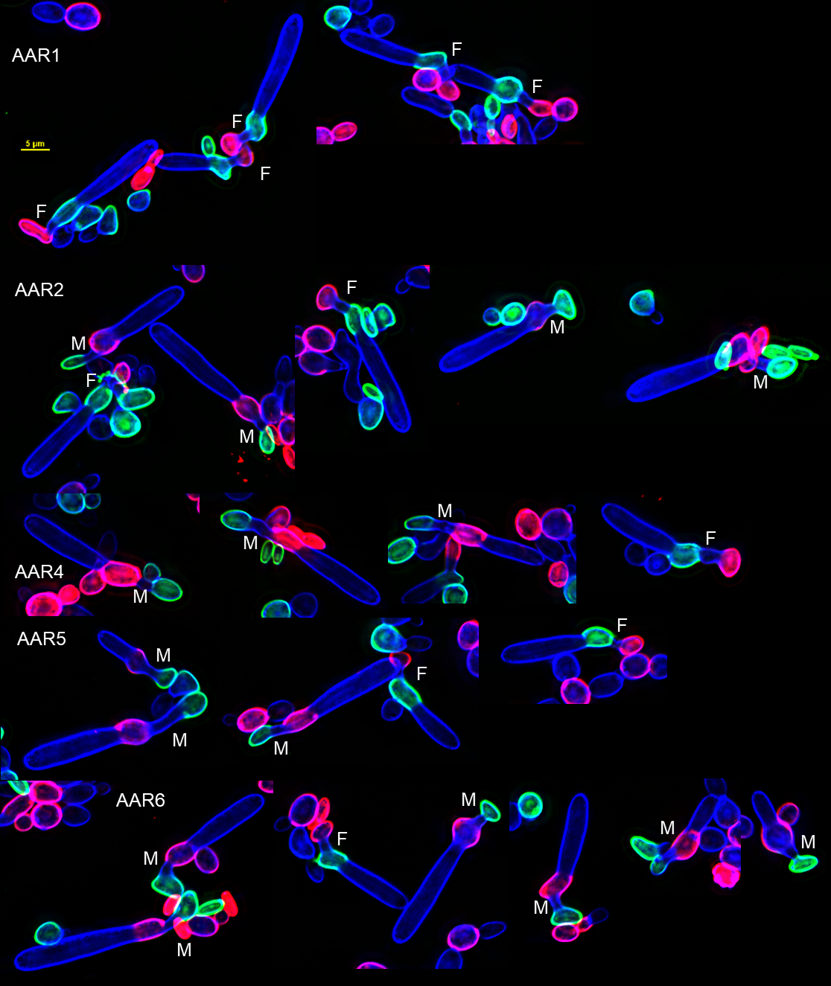

Supplement: foae014_Supplemental_Files [file foae014_supplemental_files.zip › Fig. S3 Supplementary data.tif]

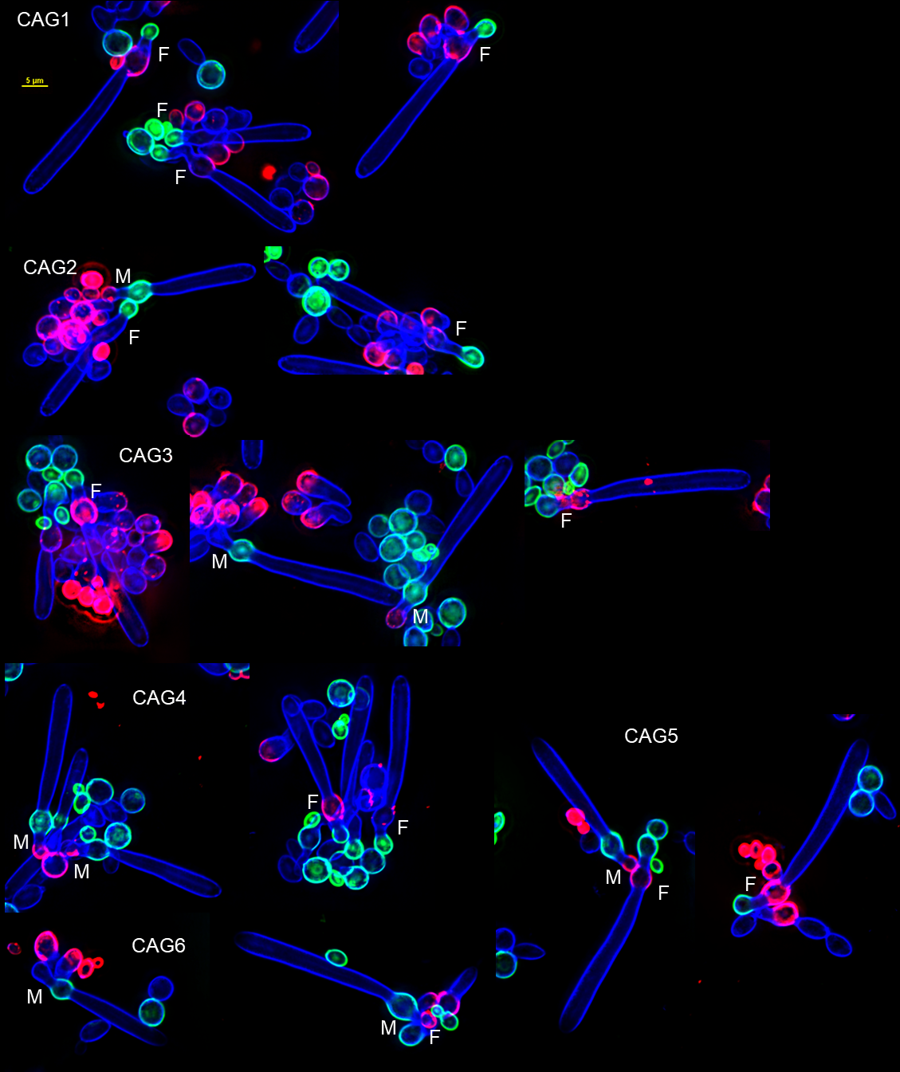

Supplement: foae014_Supplemental_Files [file foae014_supplemental_files.zip › Fig. S4 Supplementary data.tif]

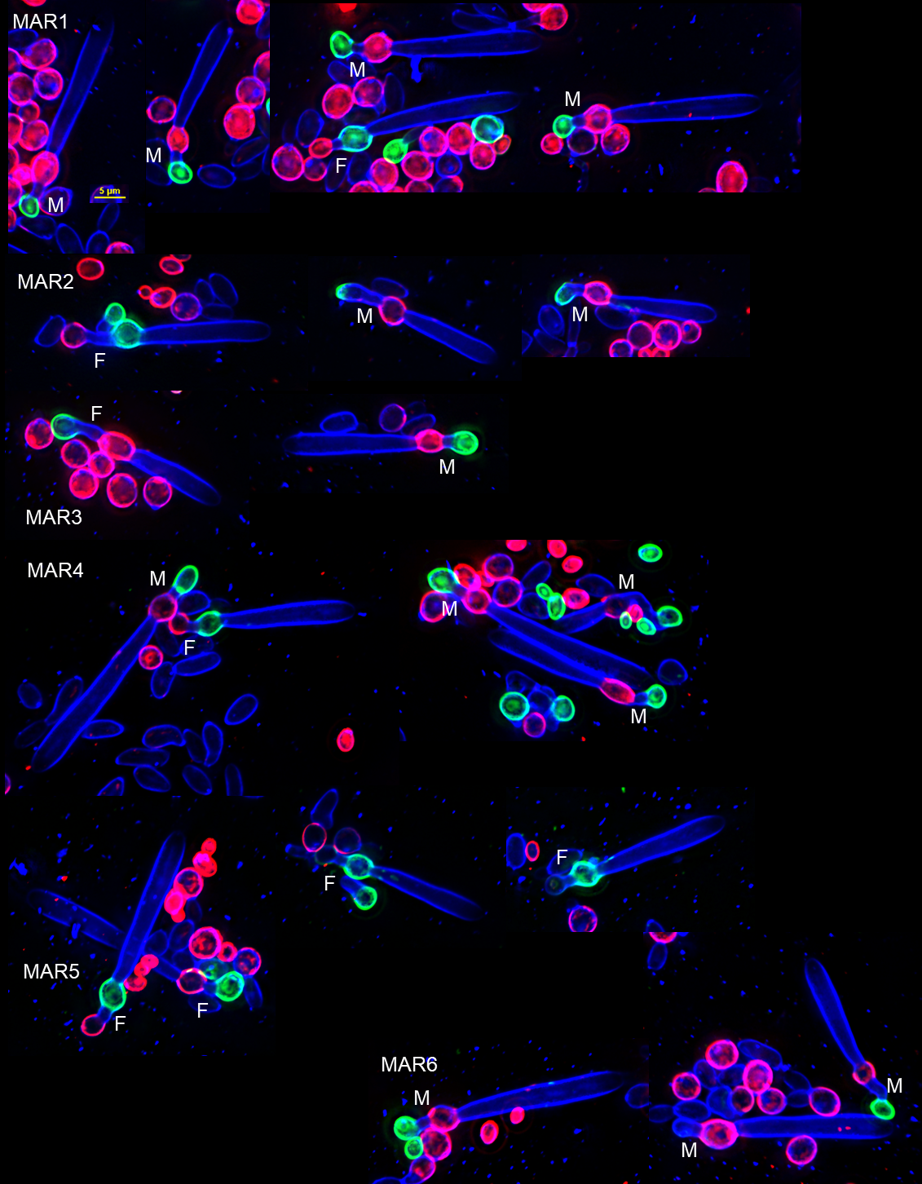

Supplement: foae014_Supplemental_Files [file foae014_supplemental_files.zip › Fig. S5 Supplementary data.tif]
